# List Decoding of Arıkan’s PAC Codes †

**DOI:** 10.3390/e23070841

**Published:** 2021-06-30

**Authors:** Hanwen Yao, Arman Fazeli, Alexander Vardy

**Affiliations:** Department of Electrical and Computer Engineering, University of California San Diego, 9500 Gilman Drive, La Jolla, CA 92093, USA; hwyao@ucsd.edu (H.Y.); afazelic@ucsd.edu (A.F.)

**Keywords:** coding theory, polar codes, convolutional codes, list decoding, sequential decoding

## Abstract

Polar coding gives rise to the first explicit family of codes that provably achieve capacity with efficient encoding and decoding for a wide range of channels. However, its performance at short blocklengths under standard successive cancellation decoding is far from optimal. A well-known way to improve the performance of polar codes at short blocklengths is CRC precoding followed by successive-cancellation list decoding. This approach, along with various refinements thereof, has largely remained the state of the art in polar coding since it was introduced in 2011. Recently, Arıkan presented a new polar coding scheme, which he called polarization-adjusted convolutional (PAC) codes. At short blocklengths, such codes offer a dramatic improvement in performance as compared to CRC-aided list decoding of conventional polar codes. PAC codes are based primarily upon the following main ideas: replacing CRC codes with convolutional precoding (under appropriate rate profiling) and replacing list decoding by sequential decoding. One of our primary goals in this paper is to answer the following question: is sequential decoding essential for the superior performance of PAC codes? We show that similar performance can be achieved using list decoding when the list size *L* is moderately large (say, L⩾128). List decoding has distinct advantages over sequential decoding in certain scenarios, such as low-SNR regimes or situations where the worst-case complexity/latency is the primary constraint. Another objective is to provide some insights into the remarkable performance of PAC codes. We first observe that both sequential decoding and list decoding of PAC codes closely match ML decoding thereof. We then estimate the number of low weight codewords in PAC codes, and use these estimates to approximate the union bound on their performance. These results indicate that PAC codes are superior to both polar codes and Reed–Muller codes. We also consider random time-varying convolutional precoding for PAC codes, and observe that this scheme achieves the same superior performance with constraint length as low as ν=2.

## 1. Introduction

Polar coding, pioneered by Arıkan [1], gives rise to the first explicit family of codes that provably achieve capacity for a wide range of channels with efficient encoding and decoding. However, it is well known that at short block lengths the performance of polar codes is far from optimal.

**Figure 1:**
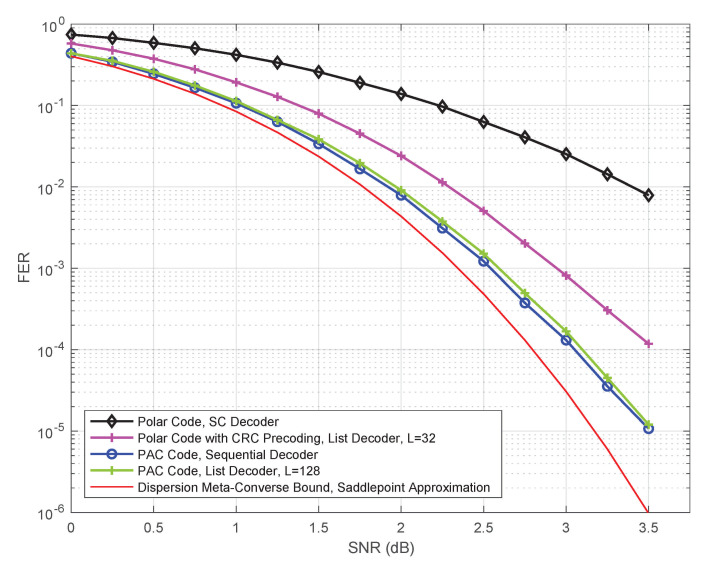
Performance of PAC codes versus polar codes.

For example, the performance of a polar code of length 128 and rate 1/2 on the binary-input AWGN channel under standard successive cancellation (SC) decoding is shown in Figure 1. Figure 1 largely reproduces the simulation results presented by Arıkan in [2]. Codes of length 128 and rate 1/2 serve as the running example throughout Arıkan’s recent paper [2], and we will also adopt this strategy herein. We make no attempt to optimize these codes; rather, our goal is to follow Arıkan [2] as closely as possible. Also shown in Figure 1 is the BIAWGN dispersion bound approximation for such codes. This can be thought of as an estimate of the performance of random codes under ML decoding (see [3]). Clearly, at length 128, there is a tremendous gap between polar codes under SC decoding and the best achievable performance.

As shown in [4] and other papers, the reasons for this gap are two-fold: the polar code itself is weak at such short lengths and SC decoding is weak in comparison with ML decoding. A well-known way to address both problems is CRC precoding followed by successive-cancellation list (SCL) decoding. Following [2], the performance of CRC-aided polar codes (with 8-bit CRC) of rate 1/2 under SCL decoding with list-size 32 is also shown in Figure 1. This approach, along with various refinements thereof (see [5,6,7] and other papers), has largely remained the state of the art in polar coding since it was first introduced in [4]. It is currently used as the coding scheme for control and physical broadcast channels in the enhanced mobile broadband (eMBB) mode and the ultra-reliable low latency communications (URLLC) mode of the fifth generation (5G) wireless communications standard [8].

In the Shannon Lecture at the ISIT in 2019, Erdal Arıkan presented a significant breakthrough in polar coding, which significantly boosts the performance of polar codes at short lengths. Specifically, Arıkan [2] proposed a new polar coding scheme, which he calls polarization-adjusted convolutional (PAC) codes. Remarkably, under sequential decoding, the performance of PAC codes is very close to the BIAWGN dispersion bound approximation [3,9]. The performance of PAC codes of length 128 and rate 1/2 is also shown (in blue and green) in Figure 1.

### 1.1. Brief Overview of PAC Codes

**Figure 2:**
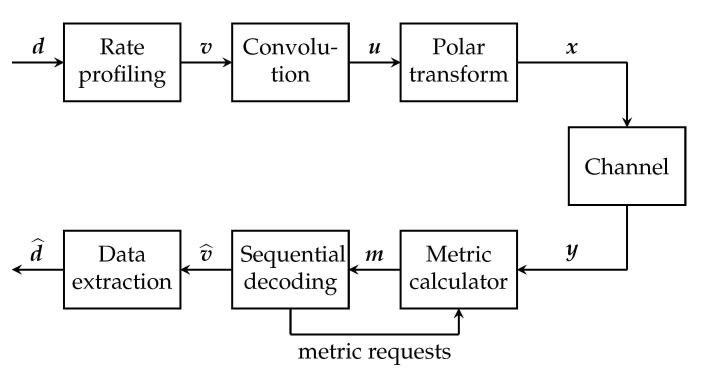
PAC coding scheme.

Arıkan’s PAC codes are largely based upon the following two innovations: replacing CRC precoding with convolutional precoding (under appropriate rate-profiling, discussed later in this section) and replacing list decoding by sequential decoding. The encoding and decoding of PAC codes are shown schematically in Figure 2, which is reproduced from [2].

Referring to Figure 2, let us consider an (n,k) PAC code. On the encoding side, Arıkan uses a rate-1 convolutional precoder concatenated with a standard polar encoder. Only *k* out of the *n* bits of the input *v* to the convolutional precoder carry the information (or data) vector *d*. The remaining n−k bits of *v* are set to 0. Just like for conventional polar codes, the overall performance of the resulting PAC code crucially depends upon which positions in *v* carry information and which are frozen to 0. This choice of frozen positions in *v*, Arıkan has termed ***rate-profiling***. Unlike conventional polar codes, the optimal rate-profiling choice is not known. In fact, it is not even clear what optimization criterion should govern this choice, although we hope to shed some light on this in Section 5.

The main operation on the decoder side is sequential decoding. Specifically, Arıkan employs Fano decoding (as described in [10] and in Section 6.9 of [11]) of the convolutional code to estimate its input *v*. The path metrics used by this sequential decoder are obtained via repeated calls to the successive-cancellation decoder for the underlying polar code.

### 1.2. Our Contributions

One of our main goals in this paper is to answer the following question: is sequential decoding essential for the superior performance of PAC codes? Is it possible, or perhaps advantageous, to replace the sequential decoder in Figure 2 by an alternative decoding method? We show that, indeed, similar performance can be achieved using list decoding, provided the list size *L* is moderately large. This conclusion is illustrated in Figure 1, where we use a list of size L=128 to closely match the performance of the sequential decoder. It remains to be seen which of the two approaches is advantageous in terms of complexity. While a comprehensive answer to this question would require implementation in hardware, we carry out a qualitative complexity comparison in  Section 4. This comparison indicates that list decoding has distinct advantages over sequential decoding in certain scenarios. In particular, list decoding is certainly advantageous in low-SNR regimes or in situations where the worst-case complexity/latency is the primary constraint.

Another objective of this paper is to provide some insights into the remarkable performance of PAC codes observed in simulations. Although theoretical analysis of list decoding remains an open problem even for conventional polar codes, it has been observed in numerous studies that list decoding quickly approaches the performance of maximum-likelihood decoding with increasing list size *L*. As expected, we find this to be the case for PAC codes as well (see Figure 7). Fortunately, maximum-likelihood decoding of linear codes is reasonably well understood: its performance is governed by their weight distribution, and can be well approximated by the union bound, especially at high SNRs. Motivated by this observation, we use the method of [5] to estimate the number of low-weight codewords in PAC codes, under polar and RM rate profiles (introduced by Arıkan [2]). We find that PAC codes with the RM rate-profile are superior to both polar codes (with or without CRC precoding) and the (128,64,16) Reed–Muller code. For more on this, see Table 3 and Figure 9 and Figure 10. We also introduce and study random time-varying convolutional precoding for PAC codes. We find that, as compared with the convolutional precoding introduced in [2], time-varying convolutional precoding is much less sensitive to the constraint length. Arıkan uses in [2] a convolutional code generated by c=(1,0,1,1,0,1,1), whose constraint length is ν=6. In Figure 12, we observe that under list decoding, random time-varying precoding achieves essentially the same performance with constraint length ν=2.

### 1.3. Related Work

Numerous attempts have been made to improve the performance of polar codes at short block lengths. Various approaches based on replacing successive-cancellation decoding with more advanced decoders include list decoding [4], adaptive list decoding [5], sequential decoding [6,12], and stack decoding [7], among others. When concatenating a polar code with an outer code, most of the existing work still uses CRC outer codes and their variants, as originally proposed in [4]. However, many other modifications of the basic polar-coding paradigm have been extensively studied, including large polarization kernels [13,14,15,16,17,18,19,20,21], polar subcodes [17,22,23,24,25,26,27], “convolutional” polar codes [28,29,30,31], and polarized Reed–Muller coding [32,33,34,35] among others.

As shown later in this paper, in Arıkan’s PAC codes, convolutional precoding combined with rate-profiling can be regarded as replacing traditional frozen bits with dynamically frozen bits. Polar coding with dynamically frozen bits was first introduced by Trifonov and Miloslavskaya in [25], and later studied in [17,22,23,25,26,27,36,37,38] and other papers. However, the dynamic freezing patterns in these papers are very different from [2]. Prior to Arıkan’s work [2], convolutional precoding of polar codes was proposed in [39] and later studied in [40].   

Although quite recent, Arıkan’s PAC codes have already attracted considerable interest; see for example [41,42,43,44,45,46,47]. While these papers investigate various aspects of PAC codes, none of them considers list decoding thereof. Finally, we note the work of [48,49], which investigates both Fano decoding and list decoding of PAC codes. This work is apparently independent from and contemporaneous with our results herein. The paper of Rowshan, Burg, and Viterbo [48] was posted on arxiv.org in February 2020, while our work [50] was submitted for review in January 2020. Our results became available on arxiv.org in May 2020. The Rowshan-Viterbo paper [49] was posted on arxiv.org in July 2020, after our results were presented in [50].

### 1.4. Paper Outline

The rest of this paper is organized as follows. We begin with an overview on Arıkan’s PAC codes in Section 2, including both their encoding process and sequential decoding. In Section 3, we present our list-decoding algorithm. In Section 4, we compare it with sequential decoding, in terms of both performance and complexity. In Section 5, we endeavor to acquire some insight into the remarkable performance of PAC codes. First, we show empirically that both sequential decoding and list decoding thereof are extremely close to the ML decoding performance. To get a handle on the latter, we estimate the number of low-weight codewords in PAC codes (and polar codes) under different rate profiles. This makes it possible to approximate the performance of ML decoding with a union bound. In Section 6, we introduce and study random time-varying convolutional precoding for PAC codes, and show that it may be advantageous in terms of the constraint length. We conclude with a brief discussion in Section 7.

## 2. Overview of Arıkan’s PAC Codes

For details on conventional polar codes under standard SC decoding, we refer the reader to Arıkan’s seminal paper [1]. Like polar codes, the block length *n* of a PAC code is also a power of 2. That is, $n=2^{m}$ with m⩾1. As shown in Figure 2, the encoding process for an (n,k) PAC code consists of the following three steps: rate-profiling, convolutional precoding, and polar encoding. In the first step, the *k* data (information) bits of the data vector *d* are embedded into a data-carrier vector *v* of length *n*, at *k* positions specified by an index set A⊆{0,1,…,n−1} with |A|=k. The remaining n−k positions in *v* are frozen to zero. Arıkan [2] used ***rate-profiling*** to refer to this step, along with the choice of the index set A.

Just like for polar codes, a careful choice of the index set A is crucial to achieve good performance. Arıkan has proposed in [2] two alternative approaches for selecting this set A. The first approach, called ***polar rate-profiling***, proceeds as follows. Let $W_{0},W_{1},\cdots ,W_{n-1}$ be the *n* bit-channels, defined with respect to the conventional polar code of length *n*. In polar rate-profiling, A is chosen so that $\left\{W_{i}:i\in \;A\right\}$ consists of the *k* best bit-channels in terms of their capacity. In other words, the capacities of the *k* bit-channels $\left\{W_{i}:i\in \;A\right\}$ are the *k* highest values among $I\left(W_{0}\right),I\left(W_{1}\right),\ldots ,I\left(W_{n-1}\right)$. The second approach proposed in [2] is called ***RM rate-profiling***. Let wt(i) denote the Hamming weight of the binary expansion of an index *i*. In RM rate-profiling, we simply pick the *k* indices of the highest weight, with ties resolved arbitrarily. In other words, the set {wt(i):i∈A} consists of the *k* largest values among wt(0),wt(1),…,wt(n−1). Notably, without convolutional precoding, this choice of A generates Reed–Muller codes (as subcodes of a rate-1 polar code).

In the second step, the data-carrier vector *v* resulting from the rate-profiling step is encoded using a rate-1 convolutional code generated by $c=(c_{0},c_{1},\ldots ,c_{\nu })$, with $c_{0}\;=\;c_{\nu }\;=\;1$ (the latter can be assumed without loss of generality). This produces another vector $u=(u_{0},u_{1},\ldots ,u_{n-1})$ of length *n*, where

$$u_{0}\;=\;c_{0}v_{0},\;u_{1}\;=\;c_{0}v_{1}+c_{1}v_{0},\;u_{2}\;=\;c_{0}v_{2}+c_{1}v_{1}+c_{2}v_{0},$$

and so on. In general, every bit in *u* is a linear combination of (ν+1) bits of *v* computed via the convolution operation:

(1) $$u_{i}\;=\;\sum_{j=0}^{\nu }c_{j}v_{i-j}$$

where for i−j<0, we set $v_{i-j}=0$ by convention. Alternatively, this step can be viewed as a vector-matrix multiplication u=vT, where T is the upper-triangular Toeplitz matrix:

(2) T=c0c1c2⋯cν0⋯00c0c1c2⋯cν⋮00c0c1⋱⋯cν⋮⋮0⋱⋱⋱⋱⋮⋮⋱⋱⋱⋱⋱0⋮⋮⋱0c0c1c2⋮00c0c10⋯⋯⋯⋯00c0⋮

In the third step, the vector *u* is finally encoded by a conventional polar encoder as the codeword $x=uP_{m}$. Here

(3) $$P_{m}\;=\;B_{n}{\left[\begin{array}{cc} \;1 & 0\;\\ \;1 & 1\; \end{array}\right]}^{⊗m}$$

where $B_{n}$ is the n×n bit-reversal permutation matrix (as defined in Section VII of [1]), and $P_{m}$ is known as the polar transform matrix. Alternatively, the polar transform can be defined as in (3) but without the bit-reversal matrix $B_{n}$; this has no effect on the performance of the resulting codes.

With reference to the foregoing discussion, the PAC code in Figure 1 is obtained via RM rate-profiling using the rate-1 convolutional code generated by c=(1,0,1,1,0,1,1). This produces the (128,64) PAC code, which is the code studied by Arıkan in [2]. This specific PAC code will serve as our primary running example throughout the paper.

On the decoding side, Arıkan [2] employs sequential decoding of the underlying convolutional code to decode the data-carrier vector *v*. Under the frozen-bit constraints imposed by rate-profiling, the rate-1 convolutional code becomes an irregular tree code. There are many different variants of sequential decoding for irregular tree codes, varying in terms of both the decoding metric used and the algorithm itself. Arıkan [2] uses the Fano sequential decoder, described in [10,11]. Notably, the path metrics at the input to the sequential decoder are obtained via repeated calls to the successive-cancellation decoder for the underlying polar code, as shown in Figure 2.

## 3. List Decoding of PAC Codes

One of our main objectives herein is to determine whether sequential decoding of PAC codes (cf. Figure 2) can be replaced by list decoding. In this section, we show how list decoding of PAC codes can be implemented efficiently (see Algorithms 1 and 2). In the next section, we will consider the performance and complexity of the resulting decoder, as compared to the sequential decoder of [2].

### 3.1. PAC Codes as Polar Codes with Dynamically Frozen Bits

To achieve efficient list decoding of PAC codes, we use the list-decoding algorithm developed in [4]. The complexity of this algorithm is O(Lnlogn), where *L* is the list size. However, the algorithm of [4] decodes conventional polar codes. In order to make it possible to decode PAC codes with (a modified version of) this algorithm, we first observe that PAC codes can be regarded as polar codes with dynamically frozen bits.

Polar coding with dynamically frozen bits was first introduced by Trifonov and Miloslavskaya in [25], and later studied by the same authors in [26,27]. Let us briefly describe the general idea. In conventional polar coding, it is common practice to set all frozen bits to zero. That is, $u_{i}=0$ for all i∈F, where F⊂{0,1,…,n−1} denotes the set of frozen indices. However, this choice is arbitrary: we can set $u_{i}=1$ for some i∈F and $u_{i}=0$ for other i∈F. What matters is that the frozen bits are fixed and, therefore, known a priori to the decoder. In [25], it was further observed that in order to be known a priori to the decoder, the frozen bits do not have to be fixed. Given i∈F, we can set

(4) $$u_{i}\;=\;f_{i}\left(u_{0},u_{1},\ldots ,u_{i-1}\right)\;$$

where $f_{i}$ is a fixed Boolean function (usually, a linear function) known a priori to the decoder. For all i∈F, the decoder can then decide as follows

(5) $${\hat{u}}_{i}\;=\;f_{i}\left({\hat{u}}_{0},{\hat{u}}_{1},\ldots ,{\hat{u}}_{i-1}\right)$$

where ${\hat{u}}_{0},{\hat{u}}_{1},\ldots ,{\hat{u}}_{i-1}$ are its earlier decisions. The encoding/decoding process in (4) and (5) is known as dynamic freezing.

In order to explain how Arıkan’s PAC codes [2] fit into the dynamic freezing framework, let us first introduce some notation. With reference to Section 2, for i=0,1,…,n−1, let $u_{i}$ and $v_{i}$ denote the vectors $\left(u_{0},u_{1},\ldots ,u_{i}\right)$ and $\left(v_{0},v_{1},\ldots ,v_{i}\right)$, respectively. Further, let $T_{i,j}$ denote the submatrix of the Toepliz matrix T in (2), consisting of the first (topmost) i+1 rows and the first (leftmost) j+1 columns. With this, it is easy to see that $u_{i}=v_{i}T_{i,i}$ for all *i*. The matrix $T_{i,i}$ is upper triangular with $\det T_{i,i}=c_{0}=1$. Hence it is invertible, and we have $v_{i}=u_{i}T_{i,i}^{-1}$ for all *i*. Now suppose that $i\in \;A^{c}$, so that $v_{i}$ is frozen to zero in the rate-profiling step. Then we have

(6) $$u_{i}\;=\;v_{i-1}T_{i-1,i}\;=\;\left(u_{i-1}T_{i-1,i-1}^{-1}\right)T_{i-1,i}$$

In particular, this means that the last bit $u_{i}$ of the vector $u_{i}$ is an a priori fixed linear function of its first *i* bits, as follows:

$$u_{i}\;=\;\left(u_{0},u_{1},\ldots ,u_{i-1}\right)\;T_{i-1,i-1}^{-1}{\left(0,\ldots ,0,c_{\nu },c_{\nu -1},\ldots ,c_{1}\right)}^{t}$$

where ${\left(0,\ldots ,0,c_{\nu },c_{\nu -1},\ldots ,c_{1}\right)}^{t}$ represents the last column of the matrix $T_{i-1,i}$. Clearly, the above is a special case of dynamic freezing in (4).

Moreover, it follows that the set F of indices that are dynamically frozen is precisely the same as in the rate-profiling step, that is $F=A^{c}$.

If i∈A, then $v_{i}$ is an information bit, but the value of $u_{i}$ is determined not only by $v_{i}$ but by $v_{i-1},v_{i-2},\ldots ,v_{i-\nu }$ as well. Thus, when representing PAC codes as polar codes, the information bits may be also regarded as dynamic.

Finally, note that in implementing the PAC decoder, there is no need to actually invert a matrix as in (6). Instead, we can successively compute the vector $\hat{v}=\left({\hat{v}}_{0},{\hat{v}}_{1},\ldots ,{\hat{v}}_{n-1}\right)$ as follows. If $i\;\in \;A^{c}$, set ${\hat{v}}_{i}=0$. Otherwise, set

(7) $${\hat{v}}_{i}\;=\;{\hat{u}}_{i}\;-\;\sum_{j=1}^{\nu }c_{j}{\hat{v}}_{i-j}$$

where the value of ${\hat{u}}_{i}$ is provided by the polar decoder. Given ${\hat{v}}_{i},{\hat{v}}_{i-1},\ldots ,{\hat{v}}_{i-\nu }$, the values of the dynamically frozen bits ${\hat{u}}_{i}$ for $i\;\in \;A^{c}$ can be computed using (1). This computation, along with the one in (7), takes time linear in ν. All that is required is additional memory to store the vector $\hat{v}=\left({\hat{v}}_{0},{\hat{v}}_{1},\ldots ,{\hat{v}}_{n-1}\right)$.
**Algorithm 1:** List Decoder for PAC Codes
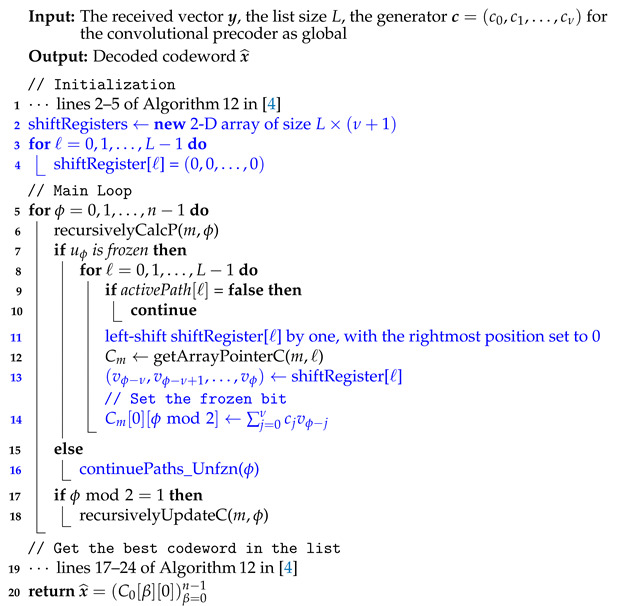


### 3.2. List Decoding of PAC Codes

Representing PAC codes as polar codes with dynamically frozen bits makes it possible to adapt existing algorithms for successive-cancellation list decoding of polar codes to decode PAC codes.
**Algorithm 2:** continuePaths_Unfzn (PAC version)
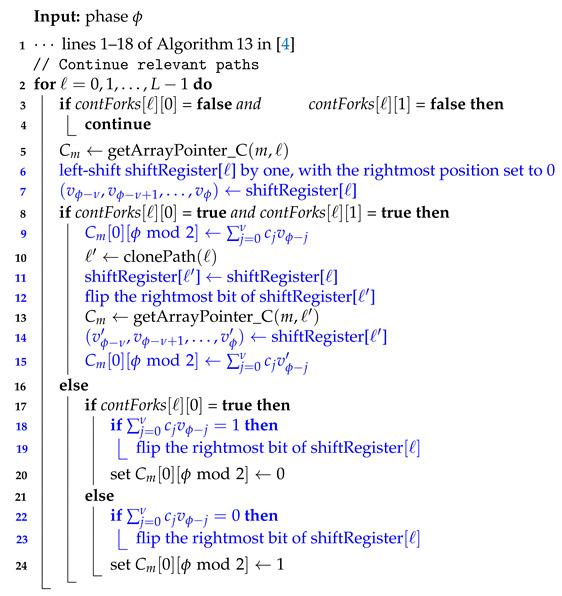


There are, however, a few important differences. For example, for conventional polar codes, when the list decoder encounters a frozen index i∈F, all the paths in the list-decoding tree are extended in the same way, by setting ${\hat{u}}_{i}=0$. For PAC codes, since freezing is dynamic, different paths are potentially extended differently, depending upon the previous decisions along the path.

In general, our list decoder for PAC codes maintains the same data structure as the successive-cancellation list decoder in [4]. In addition, for a list of size *L*, we introduce *L* auxiliary shift registers—one for each path. Each such shift register stores the last ν bits of the vector $\hat{v}=\left({\hat{v}}_{0},{\hat{v}}_{1},\ldots ,{\hat{v}}_{n-1}\right)$, computed as in (7), for the corresponding path.

Algorithms 1 and 2 provide the full details of our list decoding algorithm for PAC codes. These algorithms fit into the same general mold as Algorithms 12 and 13 of [4], with the differences highlighted in blue.

## 4. List Decoding versus Sequential Decoding

We now compare list decoding of PAC codes with sequential decoding, in terms of both performance and complexity. For list decoding, we use the algorithm of Section 3. For sequential decoding, we employ exactly the same Fano decoder that was used by Arıkan in [2]. We are grateful to Erdal Arıkan for sharing the details of their decoding algorithm. We do not disclose these details here, instead referring the reader to [2,44,45].

We note that more efficient algorithms for sequential decoding of polar codes and their subcodes may be available; see in particular the work of Trifonov [12,27]. However, in this paper, we use the results of Arıkan [2] as a benchmark, in terms of both performance and complexity. 

Our main conclusion is that sequential decoding is not essential in order to achieve the remarkable performance of PAC codes: similar performance can be obtained with list decoding, providing the list size is sufficiently large. As far as complexity, sequential decoding is generally better at high SNRs and in terms of average complexity, while list decoding is advantageous in terms of worst-case complexity and at low SNRs. 

### 4.1. Performance Comparison

**Figure 3:**
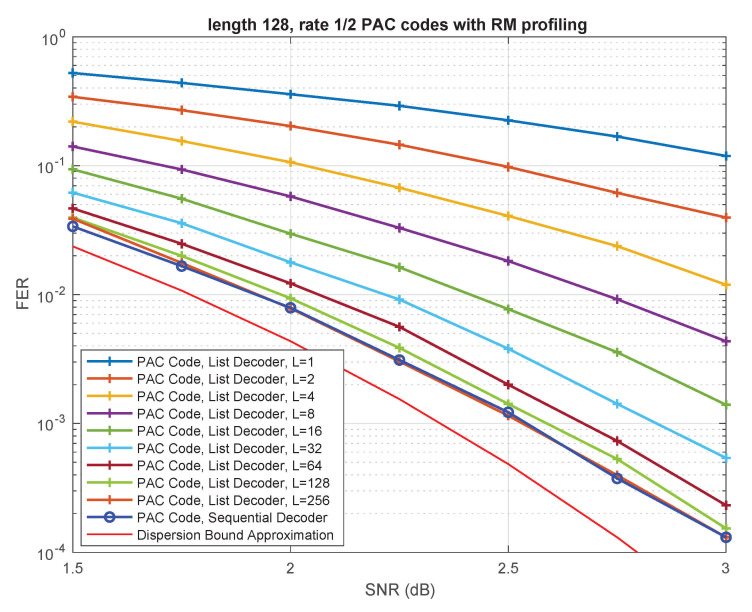
Performance of PAC codes under list decoding.

Figure 3 summarizes simulation results comparing the performance of the Fano decoder from [2] with our list decoding algorithm, as a function of the list size *L*. The underlying PAC code is the same as in Figure 1; it is the (128,64) PAC code obtained via RM rate-profiling (see Section 2). The underlying channel is the binary-input additive white Gaussian noise (BIAWGN) channel.

As expected, the performance of list decoding steadily improves with increasing list size. For L=128, the list-decoding performance is very close to that of sequential decoding, while for L=256 the two curves virtually coincide over the entire range of SNRs. 

**Figure 4:**
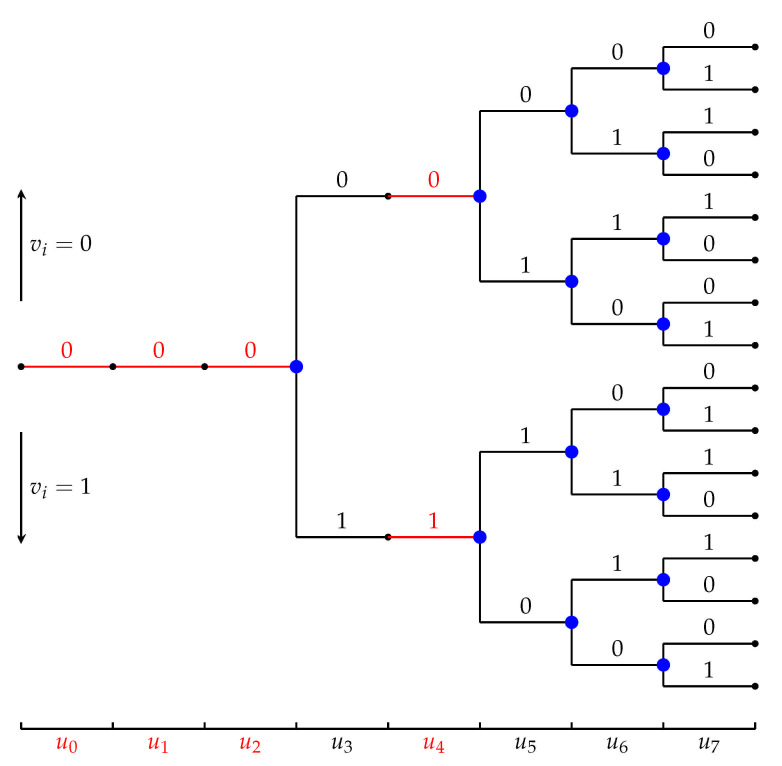
An example of the polar search tree, reproduced from [2].

It should be pointed out that the frame error rate (FER) reported for sequential decoding in Figure 1 and Figure 3 is due to two different mechanisms of error/failure. In some cases, the sequential decoder reaches the end of the search tree (see Figure 4) producing an incorrect codeword. These are decoding errors. In other cases, the end of the search tree is never reached; instead, the computation is aborted once it exceeds a predetermined cap on the number of cycles. These are decoding failures. As in [2], the FER plotted in Figure 3 counts all the cases wherein the transmitted codeword is not produced by the decoder: thus it is the sum of the error rate and the failure rate. Table 1 below shows what fraction of such cases were due to decoding failures:

A decoding failure was declared in our simulations whenever the number of cycles (loosely speaking, cycles count forward and backward movements along the search tree in the Fano decoder) exceeded 1,300,000. This is exactly the same cap on the number of cycles that was used by Arıkan in [2]. Overall, the foregoing table indicates that increasing this cap would not improve the performance significantly. In fact, we observe that decoding failures never dominate the overall FER of sequential decoding. Thus, it would be interesting to investigate how much this cap can be decreased without sacrificing the performance.

The FER for list decoding is also due to two distinct error mechanisms. In some cases, the transmitted codeword is not among the *L* codewords generated by our decoding algorithm. In other cases, it is on the list of codewords generated, but it is not the most likely among them. Since the list decoder selects the most likely codeword on the list as its ultimate output, this leads to a decoding error. We refer to such instances as selection errors. Table 2 below shows the fraction of selection errors for lists of various sizes:

This indicates that the performance of list decoding would further improve (at least, for L⩾64) if we could somehow increase the minimum distance of the underlying code, or otherwise aid the decoder in selecting from the list (e.g., with CRC).

Finally, we also include in Figure 1 and Figure 3 the BIAWGN dispersion-bound approximation for binary codes of rate 1/2 and length 128. The specific curve plotted in Figure 1 and Figure 3 is the so-called saddlepoint approximation [51] of the meta-converse dispersion bound of Polyanskiy, Poor, and Verdu [3]. Our curve coincides with those given in Figure 1 of [52] and Figure 6 of [53]. Note that a more accurate bound can be derived using the methods of Erseghe [9], but this is not critical for our purposes. It is clear from Figure 1 and Figure 3 that the performance of the (128,64) PAC code, under both sequential decoding and list decoding with L⩾128, is close to the best achievable performance.

### 4.2. Complexity Comparison

A comprehensive complexity analysis of list decoding versus sequential decoding of PAC codes in practical applications is likely to require algorithmic optimization and implementation in hardware. In the case of list decoding, this should be relatively easy based upon our representation of PAC codes as polar codes with dynamically frozen bits (see Section 3.1) in conjunction with existing work on efficient hardware implementation of polar list decoders (see [54,55], for example). On the other hand, we are not aware of any existing implementations of sequential decoding in hardware. Such implementation may be challenging due to variable running time, which depends on the channel noise, and complex control logic [56].

**Figure 5:**
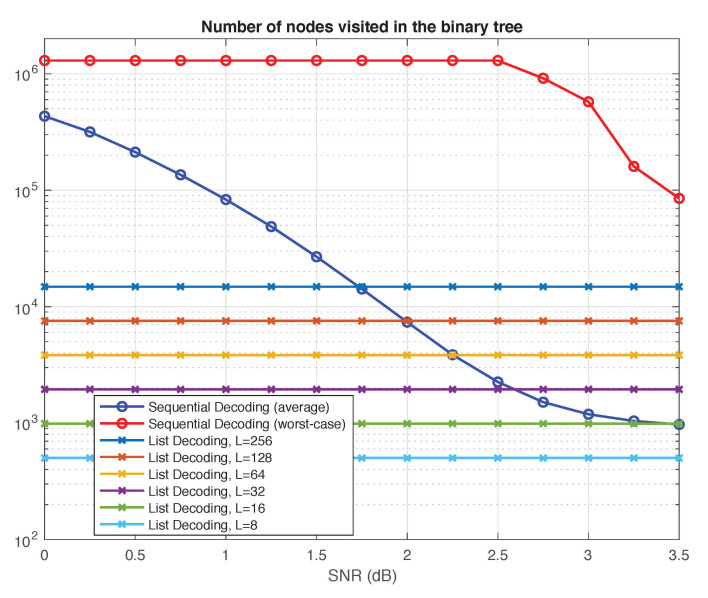
Sequential decoding vs. list decoding: Number of nodes visited in the polar search tree.

**Figure 6:**
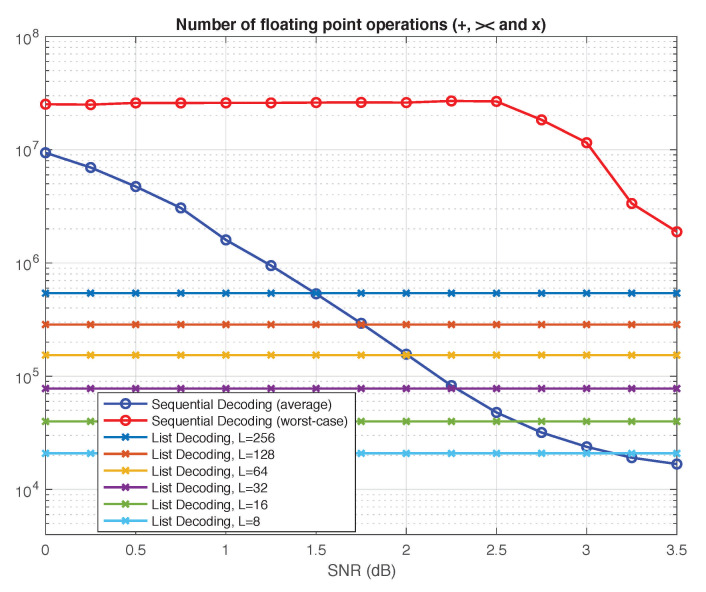
Sequential decoding vs. list decoding: Number of floating-point operations.

In this section, we provide a qualitative comparison of list decoding versus sequential decoding using two generic complexity metrics: the number of nodes visited in the polar search tree and the total number of floating-point operations performed by the decoder. The results we obtain for the two metrics, summarized in Figure 5 and Figure 6, are consistent with each other.

The polar search tree, shown schematically in Figure 4, represents all possible inputs $u=(u_{0},u_{1},\ldots ,u_{n-1})$ to the polar encoder. It is an irregular tree with n+1 levels containing $2^{k}$ paths. If $i\;\in \;\;A^{c}$ then all nodes at level *i* have a single outgoing edge, as $u_{i}$ is dynamically frozen in this case. In contrast with conventional polar codes, these edges may be labeled differently (cf. $u_{4}$ in Figure 4). If i∈A then all nodes at level *i* have two outgoing edges. In this framework, both list decoding and sequential decoding can be regarded as tree-search algorithms that try to identify the most likely path in the tree. The list decoder does so by following *L* paths in the tree, from the root to the leaves, and selecting the most likely one at the end. The Fano sequential decoder follows only one path, but has many back-and-forth movements during the decoding process.

For the sake of qualitative comparison, we take the total number of nodes the two algorithms visit in the tree as one reasonable proxy of their complexity. In doing so, we disregard the nodes at the frozen levels, counting only those nodes that have two outgoing edges (colored blue in Figure 4); we call them the decision nodes. Figure 5 shows the number of decision nodes visited by the two decoding algorithms as a function of SNR. 

For sequential decoding, two phenomena are immediately apparent from Figure 5. First, there is a tremendous gap between worst-case complexity and average complexity. For most SNRs, the worst-case complexity is dominated by decoding failures, which trigger a computational timeout upon reaching the cap on the number of cycles (see Section 4.1). Clearly, reducing this cap would also reduce the worst-case complexity. On the other hand, for SNRs higher than 2.50 dB, decoding failures were not observed. Thus, beyond 2.50 dB, the worst-case complexity gradually decreases, as expected. Another phenomenon apparent from Figure 5 is that the average complexity is highly dependent on SNR. This is natural since the processing in the Fano sequential decoder depends on the channel noise. The less noise there is, the less likely is the sequential decoder to roll back in its search for a better path.

Neither of the two phenomena above is present for list decoding: the worst-case complexity is equal to the average complexity, and both are unaffected by SNR. The resulting curves in Figure 5 and Figure 6 are flat, since the complexity of list decoding depends only on the list size *L* and the code dimension *k*.

In fact, the number of decision nodes visited by the list decoder in the polar search tree can be easily computed as follows. First assume, for simplicity, that *L* is a power of 2. As the list decoder proceeds from the root to the leaves, the number of paths it traces doubles for every i∈A until it reaches *L*. The number of decision nodes it visits during this process is given by 1+2+4+⋯+L=2L−1. After reaching *L* paths, the decoder visits *L* decision nodes at every one of the remaining $k-\log_{2}L$ levels that are not frozen. Thus, the total number of decision nodes visited is $L\left(k+2-\log_{2}L\right)-1=O\left(kL\right)$.

If *L* is not a power of 2, this counting argument readily generalizes, and the number of decision nodes visited is given by

(8) $$L\left(k+1-\lceil \log_{2}L\rceil \right)+\;2^{\left\lceil \log_{2}L\right\rceil }-1\;=\;O\left(kL\right)$$

As another qualitative metric of complexity of the two algorithms, we count the total number of additions, comparisons, and multiplications of floating-point numbers throughout the decoding process. The results of this comparison are compiled in Figure 6. The number of floating-point operations is a more precise measure of complexity than the number of decision nodes visited in the search tree. Yet we observe exactly the same pattern as in Figure 5. For list decoding, it is no longer possible to give a simple expression as in (8), but the complexity is still independent of SNR, resulting in flat curves. For sequential decoding, we again observe the same two phenomena discussed earlier in connection with Figure 5. In particular, the worst-case complexity remains prohibitive even at high SNRs.

In summary, our qualitative comparison suggests that, for a similar level of performance, sequential decoding is clearly advantageous in terms of average-case complexity at high SNRs. However, list decoding may have distinct advantages in low-SNR regimes or in situations where the worst-case complexity/latency is the primary constraint.

## 5. Performance Analysis for PAC Codes

In this section, we study the performance of PAC codes under the assumption of maximum-likelihood (ML) decoding. To this end, we estimate computationally the number of low-weight codewords in PAC codes (and other codes), then combine these estimates with the union bound. First, we explain why analysis of performance under ML decoding makes sense in our setting.

### 5.1. Sequential Decoding versus ML Decoding

It has been observed in several papers that for polar codes, list decoding rapidly approaches the performance of ML decoding with increasing list-size *L*. In this section, as expected, we find this to be the case for Arıkan’s (128,64) PAC code as well.

**Figure 7:**
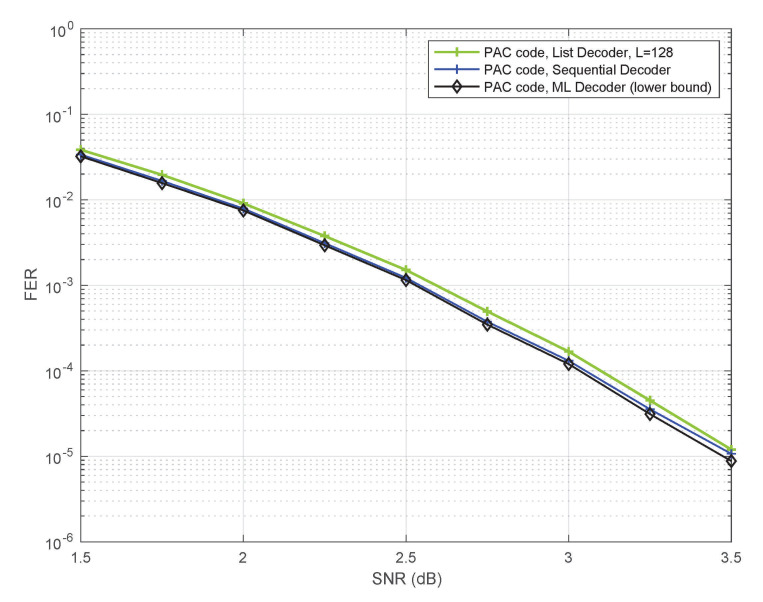
Performance of the PAC code under ML decoding.

Figure 7 shows a bound on the frame error-rate of ML decoding obtained in our simulations. This is an empirical lower bound, in the sense that the actual simulated performance of ML decoding could only be worse—even closer to the other two curves (for sequential decoding and list decoding) shown in Figure 7. The bound was generated using the Fano sequential decoder, as follows.

Every time the Fano decoder makes an error, we compare the likelihoods of the transmitted path and the path produced by the decoder. If the decoded path has a better path-metric (higher likelihood), then the ML decoder will surely make an error in this instance as well. We count such instances to generate the lower bound. This method of estimating ML performance in simulations is very similar to the one introduced in [4] for polar codes, except that [4] used list decoding. 

Figure 7 provides strong evidence that it makes sense to study PAC codes under ML decoding in order to gain insights into their performance under sequential decoding, since the two are remarkably close. Figure 7 also reveals one of the reasons why Arıkan’s PAC codes are so good at short blocklengths: they can be efficiently decoded with near-ML fidelity.

### 5.2. Weight Distributions and Union Bounds

**Figure 8:**
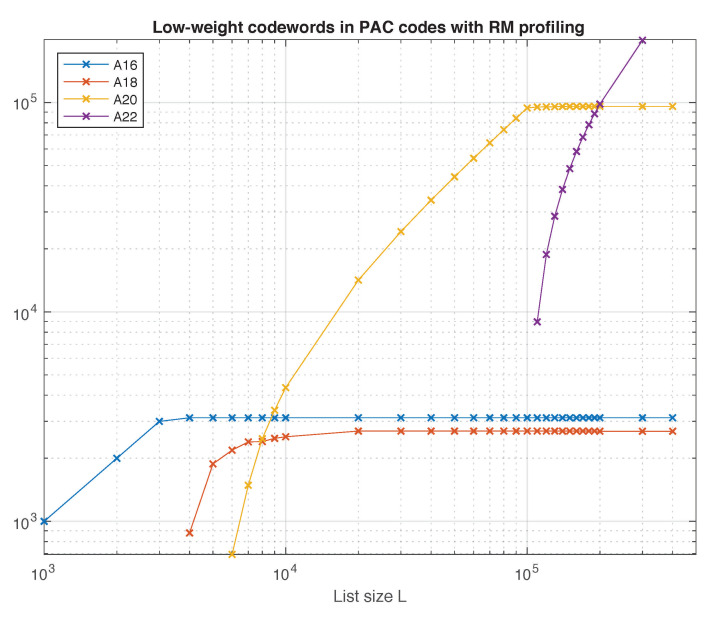
Low-weight codewords in the (128,64) PAC code.

**Figure 9:**
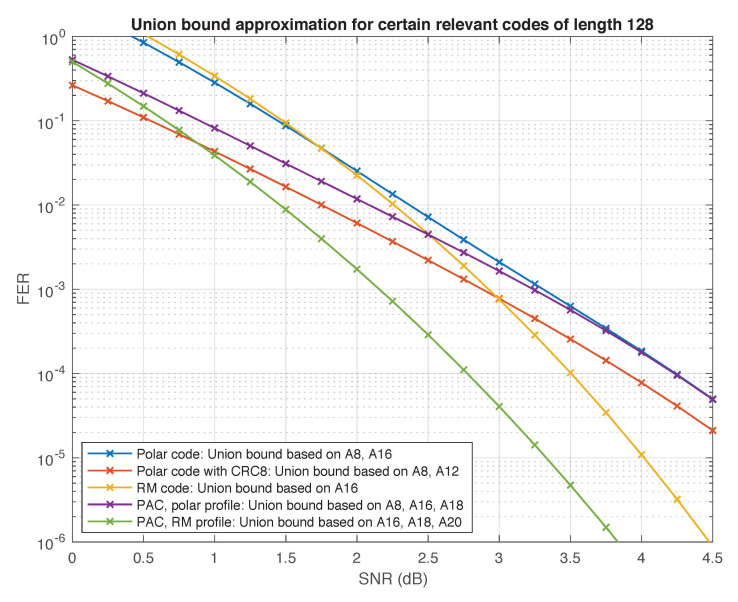
Truncated union bound for certain codes of length 128.

**Figure 10:**
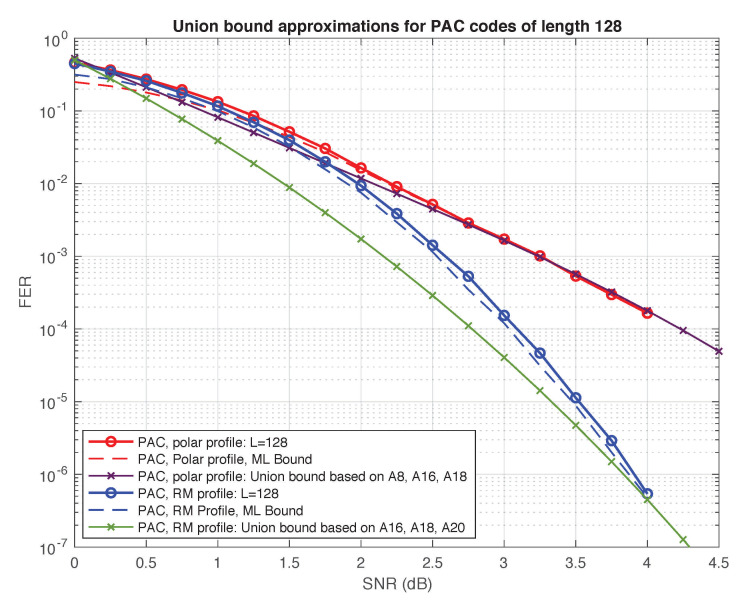
Truncated union bound vs. performance for two PAC codes.

We now study the weight distribution of the (128,64) PAC code in order to develop analytical understanding of its performance under ML decoding. The results of this study are summarized in Table 3 and in Figure 8, Figure 9 and Figure 10.

First, consider the following experiment, devised in [5]. Transmit the all-zero codeword in the extremely high SNR regime, and use list decoding to decode the channel output. It is reasonable to expect that in this situation, the list decoder will produce codewords of low weight. As *L* increases, since the decoder is forced to generate a list of size exactly *L*, more and more low-weight codewords emerge. The results of this experiment for the (128,64) PAC code are shown in Figure 8 as a function of the list size. We can see that the only weights observed for *L* up to 400,000 are 16,18,20,22. Moreover, $A_{16}⩾3120$, $A_{18}⩾2696$, and $A_{20}⩾95828$ (cf. Table 3). These numbers are lower bounds on the weight distribution of the code. However, the fact that the curves in Figure 8 saturate at these values provides strong evidence that these bounds are exact, and that codewords of other low weights do not exist.

We have used the same method to estimate the number of low-weight codewords in other relevant codes of rate 1/2, including polar codes (with and without CRC precoding), the self-dual Reed–Muller code, and the PAC code with polar rate-profile. Our results are compiled in Table 3.

Again, the numbers in Table 3 should be regarded as lower bounds, which we conjecture to be exact (except for the Reed–Muller code whose weight distribution is known [57] and the polar code whose weight distribution can be computed using the methods of [58]). Assuming this conjecture, we expect the performance under ML decoding of the (128,64) PAC code with RM rate-profile to be superior to all other polar and PAC codes in the table, since its minimum distance is twice as high. Interestingly, this code is also superior to the self-dual Reed–Muller code. The two codes have the same minimum distance, but the PAC code has significantly less codewords at this distance (by a factor of about 30). These observations are corroborated in Figure 9 and Figure 10, where we plot the truncated union bound based on the partial weight distributions compiled in Table 3 (with all other terms set to zero). It is well known that the performance of a linear code under ML decoding is governed by its weight distribution, and can be well approximated by the union bound or variants thereof [59], especially at high SNRs. The “truncated union bound” is by far the simplest option, obtained by simply ignoring those terms in the union bound for which the weight distribution is unknown. Consequently, it is neither an upper bound nor a lower bound. Nevertheless, we have found that in the high SNR regime, it provides a reasonable first-order approximation of performance under ML decoding for the codes at hand. For example, Figure 10 shows the truncated union bound for the two PAC codes in Table 3 along with upper and lower bounds on their performance (under ML decoding) obtained in simulations.

Our results in this section also provide potential guidance for the difficult problem of PAC code design. Since both sequential decoding and list decoding achieve near-ML performance, one important goal of rate-profiling should be to optimize the weight distribution at low weights. The same criterion applies for the choice of the convolutional precoder as well. A related problem is that of finding the best rate profile for a given list size, which does not necessarily approach ML decoding.

As we can see from Table 3, the (128,64) PAC code with RM rate-profile succeeds at maintaining the minimum distance d=16 of the self-dual Reed–Muller code, while “shifting” most of the codewords of weight 16 to higher weights. This is another reason for the remarkable performance of this code. The fact that the minimum distance of this PAC code is d=16 also follows from Theorem 1 of [42]. In fact, the work of Li, Zhang, and Gu [42] shows that precoding with any nonsingular upper-triangular matrix, not necessarily a Toepliz matrix as in (2), cannot decrease the minimum distance. Moreover, there always exist such upper-triangular precoding matrices that strictly reduce the number of mimimum-weight codewords (see Theorem 2 of [42]). Apparently, the Toepliz matrix generated by c=(1,0,1,1,0,1,1) is a particularly “nice” choice, reducing $A_{16}$ from 94488 to only 3120. As we shall see in the next section, there are many such “nice” matrices, and it is possible to do even better.

## 6. PAC Codes with Random Time-Varying Convolutional Precoding

With reference to Section 2, the two main considerations when designing the rate-1 convolutional precoder are: the constraint length ν and the choice of the generator $c=(c_{0},c_{1},\ldots ,c_{\nu })$. Arıkan [2] refers to such generator *c* as the ***impulse response*** of the convolutional precoder. He furthermore writes in [2] that:

*As long as the constraint length of the convolution is sufficiently large, choosing c at random may be an acceptable design practice.*

The main question we wish to address herein is this: How large is “sufficiently large” in this context? It appears that if the impulse response *c* is fixed, then constraint length on the order of ν=6 is required. However, if we allow the impulse response to vary with time, then essentially the same performance can be achieved with constraint length as low as ν=2 (which is the minimum possible, since $c_{0}=c_{\nu }=1$ by assumption). This observation is of importance if trellis methods (such as list-Viterbi decoding, as suggested in [2,49]) are used to decode PAC codes. Indeed, reducing the constraint length from ν=6 to ν=2 reduces the number of states in the resulting trellis from 64 to 4, respectively.

We also observe that under random time-varying convolutional precoding, the performance of PAC codes improves with constraint length but only slightly.

### 6.1. Random Time-Varying Convolutional Precoding

In time-varying convolutional precoding, the impulse response *c* is a function of time. Specifically, we keep the constraint length ν fixed, but use *n* potentially different impulse response vectors $c^{i}=\left(c_{0}^{i},c_{1}^{i},\ldots ,c_{\nu }^{i}\right)$, where $c_{0}^{i}=c_{\nu }^{i}=1$ for all *i*. Thus, each bit $u_{i}$ of the input $u=(u_{0},u_{1},\cdots ,u_{n-1})$ to the polar encoder is computed via a potentially different convolution operation:

(9) $$u_{i}\;=\;\sum_{j=0}^{\nu }c_{j}^{i-j}\;v_{i-j}\;\mathrm{for}\;i=0,1,\cdots ,n-1\;$$

where *v* is the data-carrier vector resulting from the rate-profiling step, as in Section 2. As before, the convolution operations in (9) can be recast a vector-matrix multiplication u=vT, where T is the following upper-triangular matrix:

(10) T=c00c10c20⋯cν00⋯00c01c11c21⋯cν1⋮00c02c12⋱⋯cν2⋮⋮0⋱⋱⋱⋱⋮⋮⋱⋱⋱⋱⋱0⋮⋮⋱0c0n−3c1n−3c2n−3⋮00c0n−2c1n−20⋯⋯⋯⋯00c0n−1⋮

In (10), the 2n−ν bits shown in red, namely $c_{0}^{i}$ and $c_{\nu }^{i}$, are set to 1, whereas the (2n−ν)(ν−1)/2 bits shown in blue are unconstrained. In what follows, we consider random time-varying convolutional precoding, where these unconstrained bits are i.i.d. $\mathrm{Bernoulli}\left(1\;/\;2\right)$ random variables. That is, each of these (2n−ν)(ν−1)/2 bits is set to 0 or 1 with probability 1/2, independently of each other.

On the decoder side, we use a straightforward modification of the list-decoding algorithm introduced in Section 3. With reference to the pseudocode in Section 3, this modification consists of replacing $c_{j}$ by $c_{j}^{\phi -j}$ at line 14 of Algorithm 1 as well as lines 9, 15, 18, 22 of Algorithm 2, where $c_{0}^{\phi },c_{1}^{\phi -1},\ldots ,c_{\nu }^{\phi -\nu }$ are as defined in (10). The complexity of such modified list-decoding algorithm is exactly the same as before; the only difference being that the decoder now needs to store the *n* impulse responses $c^{0}\;,c^{1},\;\ldots ,\;c^{n-1}$. However, this storage requirement is still linear in *n*.

### 6.2. Performance of PAC Codes with Random Time-Varying Convolutional Precoding

We now assess the performance of random time-varying convolutional precoding using our running example: the (128,64) PAC code with RM rate profile. As the comparison benchmark, we employ the convolutional precoder with ν=6 and c=(1,0,1,1,0,1,1) used by Arıkan in [2].

**Figure 11:**
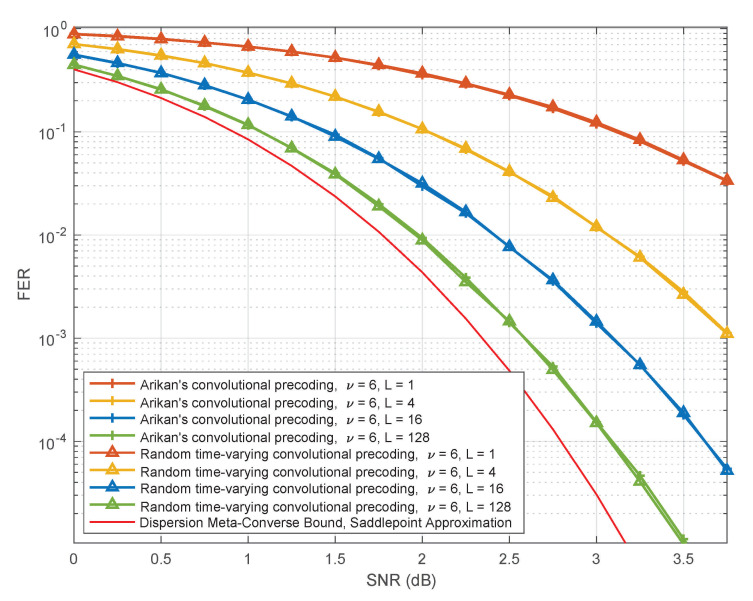
Performance of PAC codes for some specific realizations of random time-varying convolutional precoding with ν=6, as a function of the list size.

Figure 11 summarizes our simulation results for the case where the constraint length is fixed at ν=6 while the list size ranges through L=1,4,16,128. We can see from this figure that the performance of PAC codes under random time-varying convolutional precoding coincides with the list-decoding performance of the benchmark for all the relevant list sizes.

**Figure 12:**
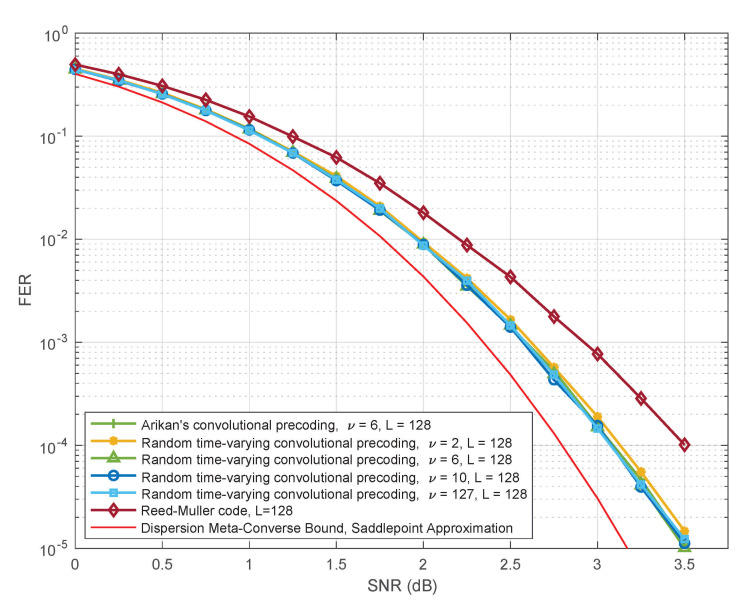
Performance of PAC codes for some specific realizations of random time-varying convolutional precoding for L=128, as a function of the constraint length.

In Figure 12, we keep the list size constant at L=128, but vary the constraint length ν. Note that setting ν=0 or ν=1 leads to degenerate cases. For ν=0, the matrix (10) reduces to the identity matrix and the PAC code reduces to the (128,64) Reed–Muller code; the performance of this Reed–Muller code is also shown in Figure 12, for comparison. For ν=1, the precoding matrix in (10) is not time-varying and not random, with each row being a shift of the vector c=(1,1). Thus the smallest nontrivial constraint length is ν=2, which allows a single bit of randomness per row in (10). Surprisingly, this suffices to closely match the performance of Arıkan’s PAC code [2] with ν=6. As we increase the constraint length in (10) beyond ν=2, the performance further improves, but very slightly. Figure 12 shows that there is no significant gain even for ν=127, in which case the precoding matrix in (10) becomes a random nonsingular upper-triangular matrix. In Table 4, we compile (lower bounds on) the weight distribution for several typical realizations of the matrix in (10) which correspond to ν=2,6,10. These results corroborate the performance observed in simulations.

## 7. Conclusions and Discussion

In this paper, we first observe that Arıkan’s PAC codes can be regarded as polar codes with dynamically frozen bits and then, using this observation, propose an efficient list decoding algorithm for PAC codes. We show that replacing sequential decoding of PAC codes by list decoding does not lead to degradation in performance, providing the list size is sufficiently large. We then carry out a qualitative complexity analysis of the two approaches, which suggests that list decoding may be advantageous in terms of worst-case complexity. We also study the performance of PAC codes (and other codes) under ML decoding by estimating the first few terms in their weight distribution. The results of this study provide constructive insights into the remarkable performance of PAC codes at short blocklengths. We furthermore introduce random time-varying convolutional precoding for PAC codes, and observe that this makes it possible to achieve the same remarkable performance with much smaller constraint length.

Based upon our results in this paper, we believe further complexity analysis of both sequential decoding and list decoding of PAC codes is warranted, including implementations in hardware. Some progress along these lines has been already reported in the recent paper [60], which uses the list-decoding algorithm introduced herein as a starting point. Indeed, we hope that our work stimulates further research in this direction.

Finally, we would like to point out two important (and interdependent) but difficult questions regarding PAC codes that remain open. What is the best choice of the rate profile? What is the best choice of the precoder? We hope our results will contribute to further study of these problems. In turn, effective resolution of these problems should make it possible to replicate the success of PAC codes at length n=128 for higher blocklengths.

## Figures and Tables

**Table 1:** Fraction of decoding failures as a function of SNR.

SNR [dB]	1.00	1.25	1.50	1.75	2.00	2.25
% of failures	4.53%	3.56%	1.86%	1.38%	1.01%	0.29%

**Table 2:** Fraction of selection errors as a function of SNR.

SNR [dB]	1.50	1.75	2.00	2.25	2.50	2.75	3.00
L=64	32.1%	32.2%	32.5%	32.3%	29.4%	36.7%	39.6%
L=128	50.0%	51.6%	54.6%	53.6%	58.4%	60.4%	63.2%
L=256	66.2%	71.0%	75.2%	78.0%	79.9%	83.6%	82.8%

**Table 3:** Number of low-weight codewords in certain relevant codes.

	$A_{8}$	$A_{12}$	$A_{16}$	$A_{18}$	$A_{20}$	$A_{22}$
Polar code	48	0	68,856	0	897,024	0
Polar code, CRC8	20	173	⩾7069	-	-	-
Reed–Muller	0	0	94,488	0	0	0
PAC, polar profile	48	0	11,032	6024	>$10^{5}$	-
PAC, RM profile	0	0	3120	2696	95,828	>10${}^{5}$

**Table 4:** Number of low-weight codewords in PAC codes for certain specific realizations of random time-varying convolutional precoding, as a function of the constraint length.

	$A_{8}$	$A_{16}$	$A_{18}$	$A_{20}$	$A_{22}$
Random precoding with ν=2	0	6424	7780	142,618	>10${}^{5}$
Arıkan’s PAC code with ν=6	0	3120	2696	95,828	>10${}^{5}$
Random precoding with ν=6	0	2870	1526	88,250	>10${}^{5}$
Random precoding with ν=10	0	2969	412	81,026	>10${}^{5}$

## Data Availability

Data is contained within the article.

## References

[1] Arıkan E. (2009). Channel polarization: A method for constructing capacity-achieving codes for symmetric binary-input memoryless channels. IEEE Trans. Inf. Theory.

[2] Arıkan E. (2019). From sequential decoding to channel polarization and back again. arXiv.

[3] Polyanskiy Y., Poor H.V., Verdu S. (2010). Channel coding rate in the finite blocklength regime. IEEE Trans. Inf. Theory.

[4] Tal I., Vardy A. (2015). List decoding of polar codes. IEEE Trans. Inf. Theory.

[5] Li B., Shen H., Tse D. (2012). An adaptive successive cancellation list decoder for polar codes with cyclic redundancy check. IEEE Commun. Lett..

[6] Miloslavskaya V., Trifonov P. (2014). Sequential decoding of polar codes. IEEE Commun. Lett..

[7] Niu K., Chen K. (2012). CRC-aided decoding of polar codes. IEEE Commun. Lett..

[8] (2020). 3GPP Technical Specification Group Radio Access Network, “Multiplexing and channel coding” Release 16, 3GPP TS 38.212 V16.3.0. https://www.etsi.org/deliver/etsi_ts/138200_138299/138212/16.03.00_60/ts_138212v160300p.pdf.

[9] Erseghe T. (2016). Coding in the finite-blocklength regime: Bounds based on Laplace integrals and their asymptotic approximations. IEEE Trans. Inf. Theory.

[10] Fano R. (1963). A heuristic discussion of probabilistic decoding. IEEE Trans. Inf. Theory.

[11] Gallager R.G. (1968). Information Theory and Reliable Communication.

[12] Trifonov P. A score function for sequential decoding of polar codes. Proceedings of the IEEE International Symposium on Information Theory.

[13] Fazeli A., Hassani H., Mondelli M., Vardy A. (2020). Binary linear codes with optimal scaling: Polar codes with large kernels. IEEE Trans. Inf. Theory.

[14] Fazeli A., Vardy A. On the scaling exponent of binary polarization kernels. Proceedings of the Allerton Conference Communication, Control, and Computing.

[15] Korada S.B., Şaşoğlu E., Urbanke R. (2010). Polar codes: Characterization of exponent, bounds, and constructions. IEEE Trans. Inf. Theory.

[16] Moskovskaya E., Trifonov P. (2020). Design of BCH polarization kernels with reduced processing complexity. IEEE Commun. Lett..

[17] Trifonov P. On construction of polar subcodes with large kernels. Proceedings of the IEEE International Symposium on Information Theory.

[18] Trifonov P. Trellis-based decoding techniques for polar codes with large kernels. Proceedings of the IEEE Information Theory Workshop.

[19] Trofimiuk G., Trifonov P. Efficient decoding of polar codes with some 16×16 kernels. Proceedings of the IEEE Information Theory Workshop.

[20] Trofimiuk G., Trifonov P. Reduced complexity window processing of binary polarization kernels. Proceedings of the IEEE International Symposium on Information Theory.

[21] Yao H., Fazeli A., Vardy A. Explicit polar codes with small scaling exponent. Proceedings of the IEEE International Symposium on Information Theory.

[22] Morozov R., Trifonov P. (2019). Successive and two-stage systematic encoding of polar subcodes. IEEE Wireless Commun. Lett..

[23] Trifonov P. Star polar subcodes. Proceedings of the IEEE Wireless Communications and Networking Conference.

[24] Trifonov P. (2020). Randomized polar subcodes with optimized error coefficient. IEEE Trans. Commun..

[25] Trifonov P., Miloslavskaya V. Polar codes with dynamic frozen symbols and their decoding by directed search. Proceedings of the IEEE Information Theory Workshop.

[26] Trifonov P., Miloslavskaya V. (2016). Polar subcodes. IEEE J. Sel. Areas Commun..

[27] Trifonov P., Trofimiuk G. A randomized construction of polar subcodes. Proceedings of the IEEE International Symposium on Information Theory.

[28] Ferris A.J., Poulin D. (2013). Branching MERA codes: A natural extension of polar codes. arXiv.

[29] Ferris A.J., Hirche D.C., Poulin D. (2017). Convolutional polar codes. arXiv.

[30] Morozov R. (2020). Convolutional polar kernels. IEEE Trans. Commun..

[31] Morozov R., Trifonov P. (2019). On distance properties of convolutional polar codes. IEEE Trans. Commun..

[32] Abbe E., Ye M. (2020). Reed-Muller codes polarize. IEEE Trans. Inf. Theory.

[33] Li B., Shen H., Tse D. (2014). RM-polar codes. arXiv.

[34] Mondelli M., Hassani S.H., Urbanke R.L. (2020). From polar to Reed–Muller codes: A technique to improve the finite-length performance. IEEE Trans. Commun..

[35] Ye M., Abbe E. (2019). Recursive projection-aggregation decoding of Reed–Muller codes. arXiv.

[36] Coşkun M.C., Neu J., Pfister H.D. Successive cancellation inactivation decoding for modified Reed–Muller and eBCH codes. Proceedings of the IEEE International Symposium on Information Theory.

[37] Miloslavskaya V., Vucetic B. (2021). Design of short polar codes for SCL decoding. IEEE Trans. Commun..

[38] Yuan P., Prinz T., Böcherer G., Iscan O., Boehnke R., Xu W. Polar code construction for list decoding. Proceedings of the 12th International ITG Conference on Systems, Communications and Coding.

[39] Fazeli A., Tian K., Vardy A. Viterbi-aided successive-cancellation decoding of polar codes. Proceedings of the IEEE Global Communications Conference.

[40] Fazeli A., Vardy A., Yao H. Convolutional decoding of polar codes. Proceedings of the IEEE International Symposium on Information Theory.

[41] Arıkan E. (2020). Systematic encoding and shortening of PAC Codes. Entropy.

[42] Li B., Zhang H., Gu J. (2019). On pre-transformed polar codes. arXiv.

[43] Mishra S.K., Kim K.C. (2020). Selectively precoded polar codes. arXiv.

[44] Moradi M., Mozammel A., Qin K., Arıkan E. (2020). Performance and complexity of sequential decoding of PAC codes. arXiv.

[45] Mozammel A. (2020). Hardware implementation of Fano decoder for PAC codes. arXiv.

[46] Tonnellier T., Gross W.J. (2020). On systematic polarization-adjusted convolutional (PAC) codes. arXiv.

[47] Wang L., Jiang M., Zhao C., Li Z. Genetic optimization of short block-length PAC codes for high capacity PHz communications. Proceedings of the International Conference on Optoelectronic and Microelectronic Technology and Application.

[48] Rowshan M., Burg A., Viterbo E. (2020). Polarization-adjusted convolutional (PAC) codes: Fano decoding vs. list decoding. arXiv.

[49] Rowshan M., Viterbo E. (2020). List Viterbi decoding of PAC codes. arXiv.

[50] Yao H., Fazeli A., Vardy A. List decoding of Arıkan’s PAC codes. Proceedings of the IEEE International Symposium on Information Theory.

[51] Vazquez-Vilar G., Fabregas A.G., Koch T., Lancho A. Saddlepoint approximation of the error probability of binary hypothesis testing. Proceedings of the IEEE International Symposium on Information Theory.

[52] Coşkun M.C., Durisi G., Jerkovits T., Liva G., Ryan W., Stein B., Steiner F. (2019). Efficient error-correcting codes in the short blocklength regime. Phys. Commun..

[53] Goldin D., Burshtein D. (2019). Performance bounds of concatenated polar coding schemes. IEEE Trans. Inf. Theory.

[54] Sarkis G., Giard P., Vardy A., Thibeault C., Gross W.J. Increasing the speed of polar list decoders. Proceedings of the IEEE Workshop on Signal Processing Systems (SiPS).

[55] Sarkis G., Giard P., Vardy A., Thibeault C., Gross W.J. (2016). Fast list decoders for polar codes. IEEE J. Sel. Areas Commun..

[56] Balatsoukas-Stimming A. (2019). Private Communication.

[57] Sugino M., Ienaga Y., Tokura N., Kasami T. (1971). Weight distribution of (128,64) Reed–Muller code. IEEE Trans. Inf. Theory.

[58] Yao H., Fazeli A., Vardy A. (2021). A deterministic algorithm for computing the weight distribution of polar codes. arXiv.

[59] Sason I., Shamai S. (2006). Performance analysis of linear codes under maximum-likelihood decoding: A tutorial. Found. Trends Commun. Inf. Theory.

[60] Zhu H., Cao Z., Zhao Y., Li D., Yang Y., Wang Y., Guo Z. (2020). Fast list decoders for polarization-adjusted convolutional (PAC) codes. arXiv.

